# Flow-Diverter Treatment for Unruptured but Growing Vertebral Artery Dissecting Aneurysm Accompanied by Simultaneous Contralateral Vertebral Artery Dissection in the Acute Phase: A Case Report and Review of the Literature

**DOI:** 10.7759/cureus.98232

**Published:** 2025-12-01

**Authors:** Yurie Rai, Satoshi Kiyofuji, Satoshi Koizumi, Yoshiaki Shiokawa, Takeo Tanishima, Akira Tamura

**Affiliations:** 1 Department of Neurosurgery, Fuji Brain Institute and Hospital, Shizuoka, JPN; 2 Department of Neurosurgery, The University of Tokyo Hospital, Tokyo, JPN

**Keywords:** bilateral vertebral artery dissection, endovascular stenting, flow-diverter stent, neurosurgery, unruptured aneurysm, vertebral artery dissecting aneurysm, vertebral dissecting aneurysm

## Abstract

Unruptured but growing vertebral artery dissecting aneurysms (VADAs) pose a significant risk of rupture with devastating outcomes. Traditional treatments, such as parent artery occlusion or trapping, with or without bypass, can be challenging when the posterior inferior cerebellar artery (PICA), anterior spinal artery (ASA), or a concurrent contralateral lesion is involved. Although flow diverters (FDs) have recently been introduced as an alternative, their efficacy, safety, and optimal timing remain unclear. A 47-year-old male presented with a headache and was diagnosed with a right VADA. Follow-up imaging over two months revealed progressive aneurysmal formation. MRI also detected a new left vertebral artery (VA) dissection without an aneurysmal change. Due to PICA and ASA involvement and the contralateral lesion, FD placement was selected to preserve the parent artery. MRI on postoperative day (POD) one demonstrated luminal thrombosis outside the FD. Digital subtraction angiography (DSA) on POD seven and six months confirmed satisfactory occlusion of the VADA with preserved PICA and ASA, as well as stability of the contralateral lesion. FD placement can be an effective treatment option for managing unruptured but enlarging VADAs, enabling parent artery preservation while avoiding compromise of a coexisting contralateral lesion. Most previous studies provide limited guidance on the optimal timing of treatment. This report highlights the effectiveness of FD placement in the acute phase to prevent rupture.

## Introduction

Intracranial vertebral artery dissecting aneurysms (VADAs) present a significant clinical challenge because of their potential to cause serious neurological events, including infarction and rupture [1,2]. The management of VADAs varies, and standardized treatment guidelines have not yet been established. Endovascular treatment with flow-diverter (FD) placement is increasingly being considered for treating VADAs, with reported complete occlusion rates ranging from 81.8% to 93.8% in the literature [3,4]. Nevertheless, the optimal timing for intervention remains uncertain. We present a case of an unruptured but growing VADA accompanied by a contralateral vertebral artery (VA) dissection treated with FD placement. A detailed chronological account of imaging findings, including MRI and digital subtraction angiography (DSA), is presented to enhance the understanding of this rare etiology. Additionally, we engage in a literature review to elucidate the optimal timing of VADA treatment.

## Case presentation

**Figure 1:**
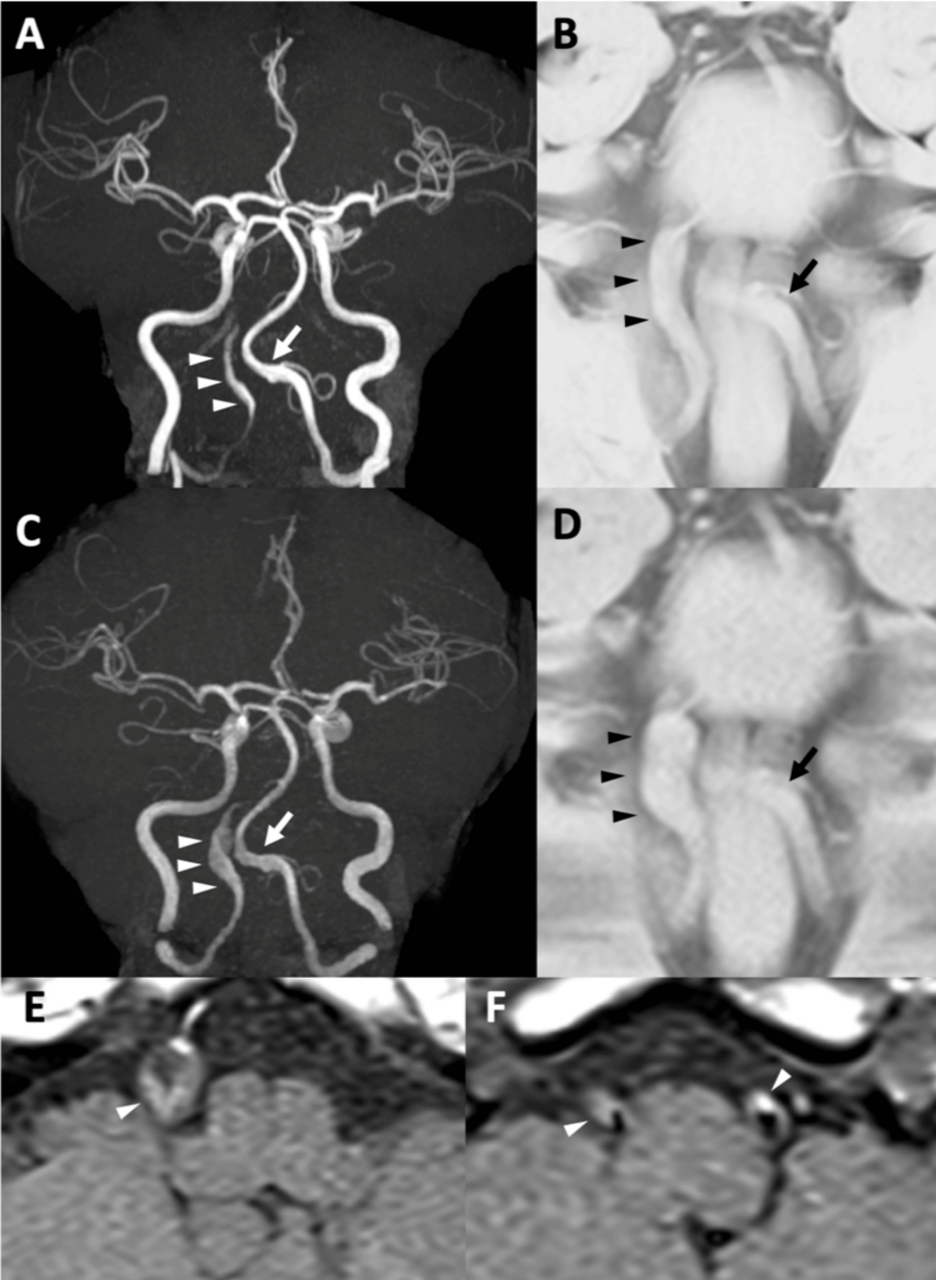
Preoperative MRA Initial images at presentation (A-B) and follow-up images at six weeks (C-F). (A) Initial MRA showed stenosis and dilation of the right V4 segment of VA (arrowheads) and the dilated left VA V4 (arrow). (B) BPAS showed the wholly dilated right VA V4 (arrowheads), contrary to MRA, and the dilated left VA V4 matching MRA (arrow). (C) MRA at six weeks showed significant enlargement of the right VADA (arrowheads) without any changes in the left (arrow). (D) BPAS showed enlargement of the right matching MRA (arrowheads) without any changes in the left (arrow). (E, F) Axial black-blood T1WI showed high-intense intramural hematoma in the bilateral VAs (arrowheads) MRA: magnetic resonance angiogram; VA: vertebral artery; BPAS: basi-parallel anatomical scanning; VADA: vertebral artery dissecting aneurysm

A 47-year-old male with a history of hyperuricemia presented with a one-week history of headache. On examination, no neurological deficits were found. Magnetic resonance angiography (MRA) revealed stenosis and dilation in the V4 segment of the right VA (Figure 1A), while basi-parallel anatomical scanning (BPAS) demonstrated diffuse dilation of the right VA (Figure 1B). T1-weighted MRI revealed high signal intensity at the affected site, suggesting intramural hematoma. No intracranial hemorrhage or infarction was detected. Based on these findings, the patient was diagnosed with an unruptured right VADA and was admitted to our hospital.

**Figure 2:**
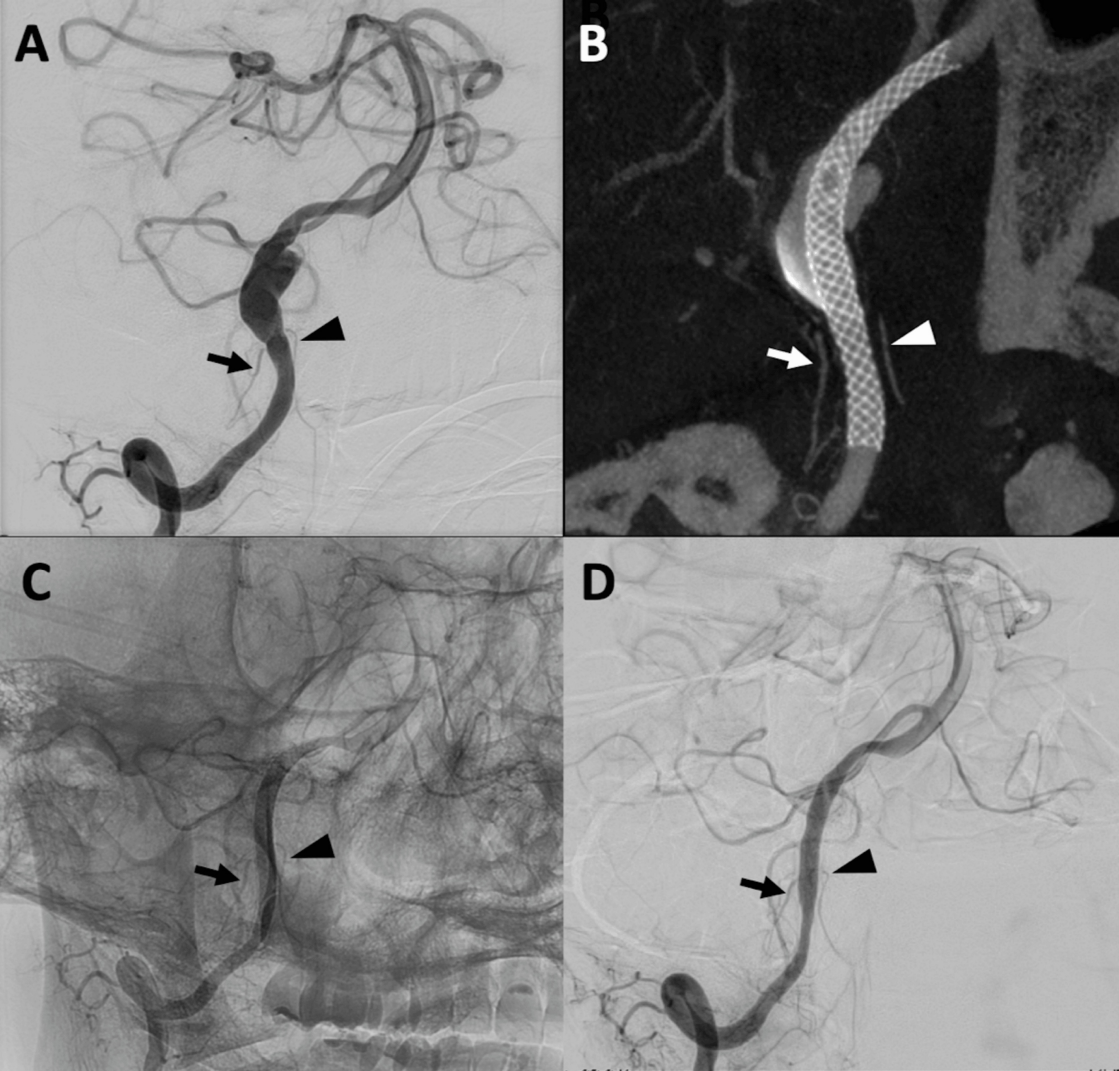
Preoperative, intraoperative, and postoperative DSA (A) Preoperative right vertebral angiogram showed the right V4 segment of VA with an irregular inner wall without stenosis, and PICA branching from the lesion (arrow) and ASA from just proximal to the lesion (arrowhead). (B) A 17% diluted contrast-enhanced 80-kV cone-beam CT immediately after the FD placement confirmed PICA (arrow) and ASA (arrowhead) patency and flow stagnation within the aneurysm and good wall apposition at PICA and ASA branches. (C) Postoperative right vertebral angiogram without subtraction on POD seven revealed complete obliteration of the aneurysm and maintained patency of PICA and ASA without in-stent stenosis. (D) Right vertebral artery angiogram at six months demonstrated complete occlusion of the VADA, while mild in-stent stenosis was noted DSA: digital subtraction angiography; VA: vertebral artery; PICA: posterior inferior cerebellar artery; FD: flow diverter; ASA: anterior spinal artery; POD: postoperative day

The patient was discharged home after conservative management with blood pressure control, and his headache resolved after five weeks. However, follow-up MRA (Figure 1C) and BPAS (Figure 1D) at six weeks showed significant enlargement of the right VADA with T1 high thrombosis (Figure 1E). Slight abnormal configuration of the left VA was noted, which was accompanied by high intensity on T1WI, suggesting a coinciding dissection of the left VA (Figure 1F). DSA revealed a dilated right V4 segment with an irregular inner wall without stenosis (Figure 2A). The posterior inferior cerebellar artery (PICA) branched just proximal to the aneurysm (Figure 2A, arrow), and the anterior spinal artery (ASA) originated from the aneurysm (Figure 2A, arrowhead). No perforators arising from the diseased segment were identified, and no collateral contributions from the contralateral ASA were observed. Parent artery occlusion or trapping was considered a high-risk treatment due to the potential for ischemia of the PICA and ASA. Furthermore, parent artery occlusion could provoke a risk of hemodynamic stress and subsequent growth of the left dissected VA. Therefore, placement of an FD, which can preserve the parent artery, was deemed the ideal treatment for this patient.

Before the treatment, dual antiplatelet therapy with aspirin 100 mg and prasugrel 3.75 mg was administered for one week. Adenosine diphosphate-induced platelet aggregation level (PAL) and collagen-induced PAL measured the day before the treatment were 2.5 and 1.6, respectively, indicating that the antithrombotic effects of both drugs were satisfactory. The procedure was performed 10 days after the detection of the aneurysmal change. Under general anesthesia, the right common femoral artery was punctured, and a 6-Fr guiding sheath FUBUKI XF 80 cm (Asahi Intecc Co Ltd, Seto, Japan) was guided into the V2 segment of the right VA following the administration of 5000 units of heparin. Phenom Plus 120 cm (Medtronic, Minneapolis, MN), Phenom 27 150 cm (Medtronic), and CHIKAI black soft tip 200 cm (Asahi) were navigated to the right VA. A three-dimensional rotational angiogram showed a maximum aneurysm diameter of 7.1 mm and a lesion length of 17 mm. A PIPELINE Flex Shield 3.5 mm x 25 mm (Medtronic) was deployed across the aneurysm. The immediate post-treatment angiogram demonstrated patent PICA and ASA, as well as flow stagnation within the aneurysm (Figure 2B). There were no periprocedural complications.

**Figure 3:**
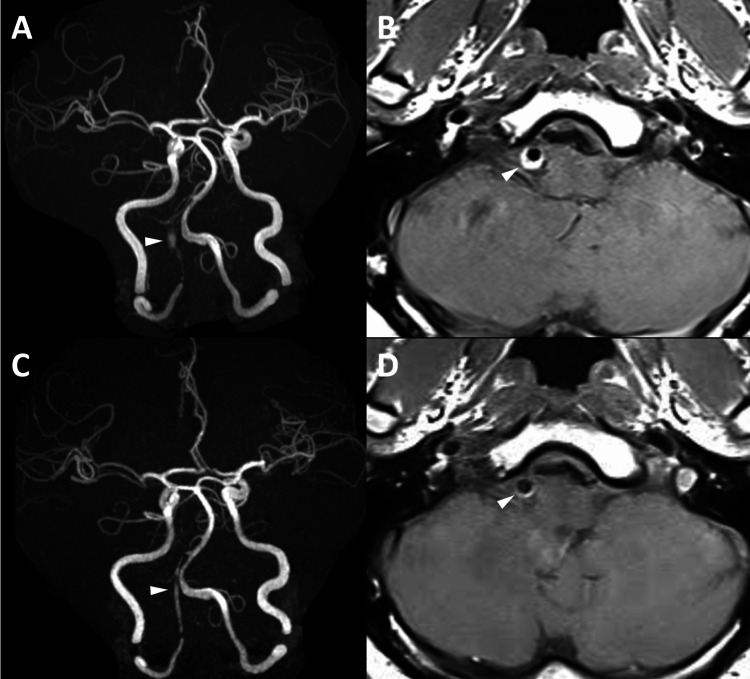
Postoperative MRA (A) MRA on POD one showed dilation of the right VA with an irregular outline (arrowhead). (B) T1WI on POD one demonstrated high intensity outside the FD (arrowhead), implicating thrombus formation outside the FD in the right VA. (C) MRA at three months demonstrated blood flow only within the FD (arrowhead). (D) T1WI at three months demonstrated a decrease in the intensity outside the FD with no neointimal hyperplasia within the FD (arrowhead) MRA: magnetic resonance angiogram; POD: postoperative day; VA: vertebral artery; FD: flow diverter

MRA on postoperative day (POD) one showed an irregular outline of the right VA, which suggested the aneurysm still had remnant intra-aneurysmal flow outside the FD (Figure 3A). T1WI demonstrated high intensity outside the FD, representing thrombus formation (Figure 3B). MRA on POD seven demonstrated blood flow only within the FD, while T1WI showed a gradual decrease in intensity outside the FD. A follow-up angiogram on POD seven revealed complete obliteration of the aneurysm with preserved patency of the PICA and ASA (Figure 2C), and the patient was discharged home without any complications on POD eight. At three months, follow-up MRA again confirmed complete aneurysm obliteration (Figure 3C), allowing discontinuation of dual antiplatelet therapy with aspirin and prasugrel and transition to aspirin monotherapy. Follow-up angiogram at six months demonstrated complete occlusion of the VADA, while mild in-stent stenosis was noted (Figure 2D). The patient has therefore remained on aspirin therapy.

## Discussion

Literature review

We performed a literature review and identified 13 studies evaluating FD treatment for VADA, summarized in Table 1 [5-17]. Most studies reported high aneurysmal occlusion rates ranging from 55.6 to 95.7%, and low complication rates between 0% to 16.6%. Twelve of the studies did not specify the timing of treatment; however, Suh et al. noted that 14 cases (60.9%) underwent FD placement during the acute or subacute phase (within three months), while nine cases were treated in the chronic phase (beyond three months) [9]. Several previous studies included VADAs located on the dominant VA and/or involving branches, highlighting the superiority of FD in preserving the parent artery and associated branches. Long et al. and Oh et al. each reported one patient with bilateral VADA treated with FD, although it remains unclear whether untreated contralateral lesions were present in the remaining cases we reviewed [6,10].

**Table 1:** Studies invovling flow diverter treatment for unruptured vertebral artery dissecting aneurysm ^*^Including O'Kelly-Marotta (OKM) grade C and D. **Including co-dominant ND: not described

Author, year	Patients, n	Age (years)	Complication, n (%)	Complete occlusion, %	Treatment timing	Dominant, n (%)	Branch involvement, n (%)	Bilateral lesion, n
Present case, 2025	1	47	0	100	Within 3 months	1 (100)	1 (100)	1
Kim et al., 2024 [5]	25	55.2	0	81.8^*^	ND	7 (28)^**^	6 (24.0)	ND
Long et al., 2024 [6]	17	ND	1 (5.8)	78.6^*^	ND	ND	ND	At least 1
Lu et al., 2023 [7]	16	55.1	0	66.7	ND	ND	3 (18.8)	ND
Han et al., 2023 [8]	65	53	3 (4.6)	87.7	ND	44 (69.8)	14 (21.5)	ND
Suh et al., 2023 [9]	23	48.5	0	95.7	14 (60.9%) within 3 months, 9 (39.1%) after 3 months	3 (13.0)	11 (47.8)	ND
Oh et al., 2022 [10]	26	55.2	2 (7.7)	55.6	ND	14 (51.9)	6 (23.1)	At least 1
Liu et al., 2022 [11]	24	ND	0	76.5	ND	ND	ND	ND
Li et al., 2022 [12]	25	52	4 (16)	57.9	ND	ND	8 (32)	ND
Lee et al., 2022 [13]	12	54.6	2 (16.6)	60	ND	ND	4 (33.3)	ND
Kim et al., 2021 [14]	9	54	1 (11.1)	66.7	ND	2 (22.2)	3 (33.3)	ND
Fu et al., 2021 [15]	32	52	0	56.7	ND	ND	32 (100)	ND
Catapano et al., 2022 [16]	22	ND	0	ND	ND	22 (100)	ND	ND
Gölitz, 2016 [17]	11	58	0	90.9	ND	ND	5 (45.5)	ND

Treatment selection of FD for VADA

VADA is a rare condition with an incidence of 2.01 per 100,000 person-years [18]. While unruptured VADAs generally have a good prognosis, complications such as stroke or hemorrhage may occur, necessitating careful follow-up and case-by-case treatment strategies [1,2].

In our case, we detected a rapid enlargement of the VADA, promoting surgical intervention. A contralateral VA dissection, which is rare with an incidence of just 0.33% among intracranial VADAs [19], further complicated the treatment decision. Previous reports indicate that VADAs may develop after the sacrifice of the contralateral VA [20-22], sometimes leading to subarachnoid hemorrhage [20,22]. This suggests that hemodynamic stress on a dissected VA, resulting from contralateral VA sacrifice, can promote enlargement and potentially fatal rupture. In the meantime, from an open microsurgical standpoint, combinations of bypass and trapping/occlusion are both safe and effective, as they maintain normal parent artery blood flow in the diseased VA [23,24]. However, in our case, even trapping or short-segment occlusion of the diseased VA itself was not feasible due to the involvement of the PICA and ASA. Consequently, we opted for FD placement to preserve the parent artery. To date, our literature review has identified 308 cases of VADAs treated with FD placement, including the present case.

Rapid occlusion of VADA after FD placement

Our case is the first to document complete obliteration of an unruptured VADA within just one week following FD placement; previously, only one case (involving a ruptured VADA) reported complete occlusion as early as five days after treatment [25]. Typically, obliteration in such cases has been assessed by DSA or CTA three months or later [3].

One theory is that the timing of FD placement may influence occlusion outcomes. In the early phase of VA dissection, active thrombus formation might occur [26,27], and in vitro experiments indicate that endothelial regeneration at the rupture site of the internal elastic lamina begins around one week, completing around three months [28]. In our case, we likely treated the patient during the acute thrombus formation phase, as demonstrated by chronological changes in preoperative and postoperative T1WI images, which may have contributed to the successful obliteration. Previous studies have not clarified the optimal treatment timing for VADAs. Our case suggests that early intervention in the acute phase may facilitate rapid aneurysm resolution.

Early aneurysm thrombosis following FD placement may offer advantages; however, caution is warranted as it may cause rupture through aggressive thrombus-associated autolysis, rather than reverse remodeling and cicatrization. A previous report showed early rupture in 10 patients post-FD placement (mean: 16 days; range: 2-­48 days), and three patients encountered rupture three to five months after FD placement, all with extensive thrombosis before rupture, although the rupture rate is unknown [29]. At the same time, the risk of in-stent stenosis or occlusion should also be noted. A previous study comparing FD and stent-assisted coiling for unruptured VADAs found higher rates of in-stent stenosis with FD [8]. For intracranial aneurysms, the risk of in-stent stenosis is reported up to 15.0% for pipeline embolization devices and 2.4% with stent-assisted coiling [30,31]. In our case, a follow-up angiogram at six months showed mild in-stent stenosis. This should be carefully monitored with an MRI.

Technical nuances of FD treatment for VADA

In addition to timing, anatomy and technique may be crucial for achieving complete obliteration without ischemic events. The VA and basilar artery have numerous perforators, and placement of an FD provokes a risk of ischemia on these vessels [32]. In our case, it was particularly important to crimp the FD at the ASA and PICA branch sites, and this was satisfactorily confirmed by post-placement cone-beam CT. Before FD placement, assessing anatomical branching near the lesion, determining the FD coverage area, and selecting the appropriate stent diameter and length are critical technical factors for successful outcomes.

## Conclusions

We presented a case of a growing VADA treated with FD, accompanied by contralateral VA dissection to preserve the parent artery, resulting in rapid and complete obliteration. FDs may play an important role in managing VADAs; however, further studies with larger sample sizes and longer follow-up are needed to establish the optimal treatment strategy for VADAs.
